# Connexins in Lung Cancer and Brain Metastasis

**DOI:** 10.3389/fonc.2020.599383

**Published:** 2020-12-23

**Authors:** Kai-Jun Luo, Chang-Xu Chen, Jia-Peng Yang, Yun-Chao Huang, Eduardo R. Cardenas, Jean X. Jiang

**Affiliations:** ^1^ School of Life Sciences, Yunnan University, Kunming, China; ^2^ Key Laboratory of the University in Yunnan Province for International Cooperation in Intercellular Communications and Regulations, Yunnan University, Kunming, China; ^3^ Department of Thoracic Surgery I, The Third Affiliated Hospital of Kunming Medical University/Yunnan Cancer Hospital, Yunnan Cancer Center, Kunming, China; ^4^ Joint International Research Laboratory of Regional Tumor in High Altitude Area, Kunming, China; ^5^ Department of Biochemistry and Structural Biology, University of Texas Health Science Center, San Antonio, TX, United States

**Keywords:** lung cancer brain metastasis, connexin, gap junction, hemichannel, endothelial cells, astrocytes

## Abstract

Connexins (Cxs) are involved in the brain metastasis of lung cancer cells. Thus, it is necessary to determine whether gap junction-forming Cxs are involved in the communication between lung cancer cells and the host cells, such as endothelial cells, forming the brain–blood-barrier, and cells in the central nervous system. Data from multiple studies support that Cxs function as tumor suppressors during lung cancer occurrence. However, recent evidence suggests that during metastasis to the brain, cancer cells establish communication with the host. This review discusses junctional or non-junctional hemichannel studies in lung cancer development and brain metastasis, highlighting important unanswered questions and controversies.

## Introduction

**Figure 1:**
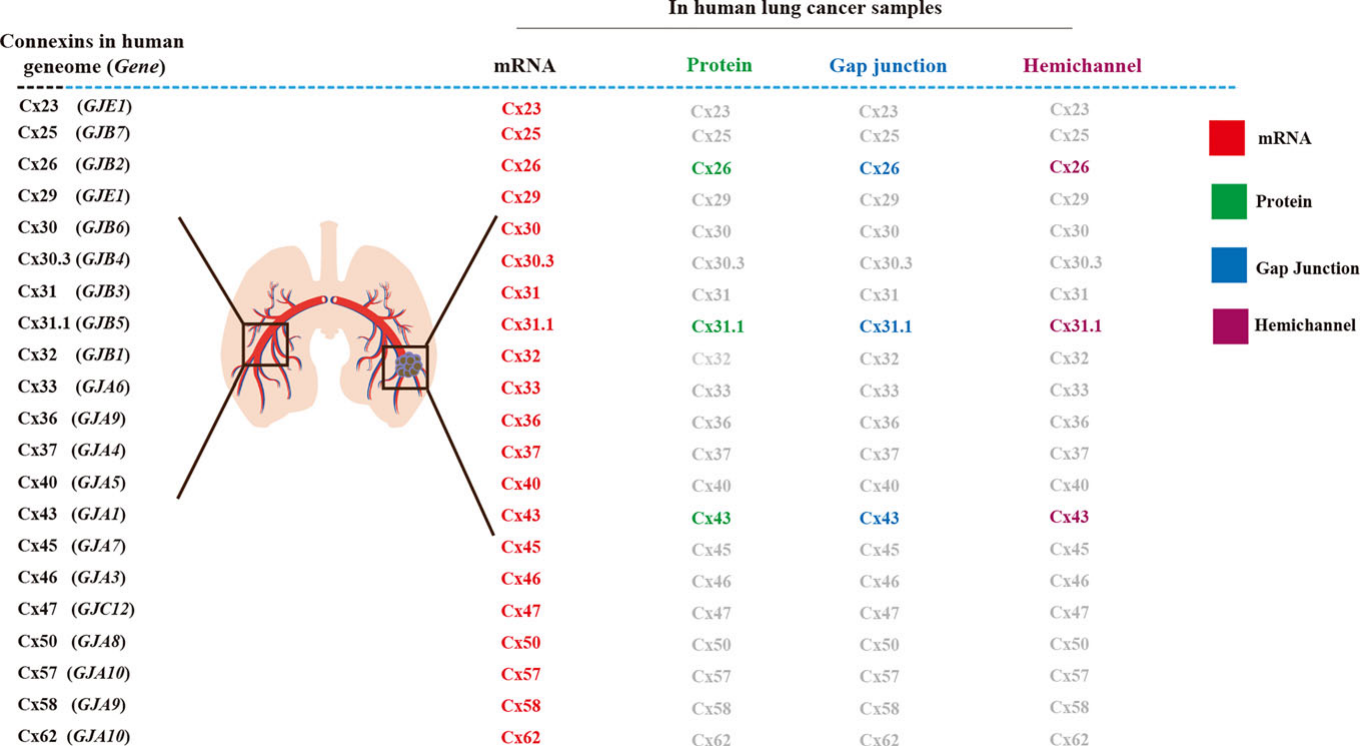
Connexins (Cxs) in human lung cancer tissue and its Cx-channels. In normal lung tissues, 21 connexin genes, mRNA, and protein have been detected in the human genome, junctional hemichannel, and hemichannels (1, 2).

The understanding of Connexin (Cx) channels in lung cancer brain metastasis is rather limited. In the human genome, 21 Cxs are found (**Figure 1**) and in the mouse genome, 20 mCx genes are found (3–6). A connexin hemichannel is also known as a connexon, and comprises a hemichannel assembly with six connexin subunits formed on the cell membrane; two hemichannels from neighboring cells dock together to form gap junctions (GJs) (7, 8). GJs are intercellular communication junctions, important in the maintenance of homeostasis, and their disruption is associated with pathology, particularly in carcinogenic processes (9, 10). GJs are responsible for intracellular communication *via* the passing of small ions and hydrophilic metabolites less than 1 kDa in size. In addition to forming gap junctions, hemichannels provide unique cell permeability between the intracellular and extracellular milieus in tissue function and affect tumorigenesis, suppression of cancer growth, and metastasis (11, 12).

Metastasis results in the dissemination of cancer cells to a new organ. Over half of the brain metastases are derived from lung adenocarcinoma. The dissemination of lung cancer cells, especially to brain parenchyma and leptomeninges, causes high morbidity; as this metastasis is primarily associated with the central nervous system, it leads to a poor prognosis (13). Lung cancer cells need to overcome several stages or barriers to metastasize to the brain. First, the cells must grow at their site of origin and detach from neighboring cells in the lung. Next, they must intravasate to nearby blood vessels, penetrating blood vessels, then cross the blood-brain barrier (diapedesis). Finally, to establish itself as a metastatic cancer cell in the brain, the cancer cells must undergo angiogenesis to form a new blood supply (14). However, the understanding of the role of Cx-channels in lung cancer brain metastasis is limited, and necessitates further research.

## Connexins, Gap Junctions, Hemichannel, and Lung Cancer Types

Different isoforms of Cx may have distinct functions in lung cancer tissues and cell lines.

Non-small cell lung cancer (NSCLC) and small cell lung cancer (SCLC) are the two foremost lung cancers; furthermore, NSCLC has been classified into three subclasses: NSCLC-la (large cell carcinoma), NSCLC-sq (squamous cell carcinoma), and NSCLC-ad (adenocarcinoma). These subclasses normally stem from the bronchioles, small alveoli, and alveoli (15).

Cx expression at the transcriptional and protein translation levels have been observed in human lung cancer tissues, using electrophysiology and dye uptake techniques, to identify Cx biomarkers for clinical prognosis. To date, 21 Cx mRNAs have been detected in lung cancer tissues including in NSCLC-ad and NSCLC-sq, with differential abundance compared to that in normal healthy lung tissues (1). Although the mRNA levels of these Cxs do not represent hemichannel functionality or gap junction intercellular communication (GJIC), researchers have demonstrated that high Cx mRNA levels are associated with better survival, suggesting the association of Cx expression with tumor-suppressive roles (1). Other indicators suggest that Cxs may also function as tumor promoters, as their expression correlates with poor survival (1). Therefore, information such as cancer stage and cancer type is key (1). Cx26 significantly correlates with poor prognosis and metastasis in NSCLC-sq (2). The protein, hemichannel, and GJ functions of Cxs, such as Cx43 (1) and Cx26 require further investigation (2) (**Figure 1**).

An interesting link between Cxs and lung cancer brain metastasis has been observed in the NSCLC-la cell line SK-LuCi-6, derived from lung cancer brain metastatic tumors, which present detectable levels of GJIC. However, other SCLC cell lines (NCI-H209, SV-E, LD-T, and MO-A) derived from metastatic carcinomas, lack GJIC (16). Similar to previous findings in human lung cancer cells, these SCLC metastatic cell lines typically exhibit low GJIC because of decreased Cx expression. SK-LuCi-6 presents higher GJIC levels, suggesting different GJIC functions in different subtypes of lung cancers.

Furthermore, different isoforms of Cxs have been directly associated with tumor progression. Ectopic expression of Cx43 in LH_7_ cell lines derived from the highly metastatic human pulmonary giant cell carcinoma, indicates low levels of Cx43 enabling reestablishment of intracellular communication, causing more normal characterization (17). Notably, in the human NSCLC-ad cells lines, HCC827 and PC9, Cx26 expression triggered EMT (epithelial–mesenchymal transition) involving the PI3K/Akt signaling pathway (18). Rodent lung cancer models have also provided valuable information on Cx association with lung cancer metastasis. In mouse lung cancer tissue, altered expression of mCx26, mCx32, mCx37, mCx40, mCx43, and mCx45 mRNA is observed during lung tumorigenesis compared to normal lung tissue (19). These mCx proteins are known to be engaged in GJIC in lung cancer. Rat Cx26 mRNA expression was reduced in lung adenocarcinomas and induced in rats using N-nitrosobis(2-hydroxypropyl)amine, suggesting rCx26 involvement in tumor development (20).

Therefore, the functional roles of Cxs are largely influenced by mechanisms, both dependent and independent of gap junctions, type of lung cancer, and stage of lung cancer progression.

## Primary Lung Cancer and Cxs Expression

### Broken Steady State of Cx43 During Lung Cancer Progression

It is reported that Cx43 serves as an inhibitor of lung tumorigenesis in the early stage; however, a recent study revealed that Cx43 can also function as a tumor promoter (1). This controversy could be attributed from the fate of Cx43 at different stages of lung cancer: early stage, before metastasis to other tissue, and in the advanced stage of primary lung cancer. Cx43 mRNA and protein can be detected in tissues obtained from early stage human lung cancer, although the expression level is lower and is nucleus-localized compared with heathy lung tissue (1). Interesting, Cx43 mRNA and protein expression are gradually decreased in normal lung tissue adjacent to the tumor tissue and was closely correlated with the distance from the tumor tissue as Cx43 expression was lower in areas closer to the tumor site (21). These data suggest that cancer cells negatively affect Cx43 expression in the surrounding normal lung cells. Moreover, it also implies that primary lung cancer cells appear to “isolate themselves” by preventing the GJICs between lung cancer cells and normal lung cells.

However, the picture is quite different in advanced lung tumor. In the advanced stage of lung tumor, Zhao et al. found that Cx43 expression is positive in human NSCLC tissue in the advanced stage of lung cancer. This study suggests that Cx43 can be an important biomarker for the progression of NSCLC from lower grades with undetectable Cx43 levels, to higher grades during metastasis with poor prognosis (22). Furthermore, the other study indicates that Cx43 can be a prognostic factor to forecast advanced NSCLC, as high Cx43 is associated with a positive prognosis; in contrast, lower Cx43 is associated poor prognosis (23). Higher Cx43 in A549 cells lead to cisplatin (cis-diaminodichloroplatinum) chemotherapy resistance and reversed the EMT (18). Alternatively, Cx43 reverses resistance against EMT inhibition by cisplatin in A549/cisplatin cells, and lower Cx43 expression was observed in A549/cisplatin cells in comparison to A549 cells. These cells acquired an EMT phenotype with morphological changes, such as spindle-like fibroblasts (21). In mCx43 heterozygous knockout mice mCx43^+/−^, mCx43 loss was associated with lung cancer aggression and a higher incidence of NSCLC-ad induced by the known carcinogen 7,12-dimethylbenzanthracene (24). The difference in expression levels may explain how Cx43 can play opposed roles as a tumor inhibitor and enhancer depending upon the stages of lung cancer development.

There are ample evidences supporting inhibitory function of Cx43 and GJIC in lung cancer cell lines and primary lung cancer growth. Using lung cancer cell line, an assay performed in an *in vitro* by Ruch et al. showed that forced Cx43 expression in a human cancer cell line restored GJIC and reduced cell growth and tumorigenicity (25). More recently, the same group reported an association between Cx43 and the neoplastic transformation of lung cancer stem cells (CSCs). Cx43 reversed some tumor features, and reduced the number of lung CSC in human (26). In the cell lines and tissue of lung tumor from mouse, GJIC is commonly defective, and the loss of GJ proteins, such as mCx43, results in the loss of a crucial component in intercellular communication and a vital intermediary of regulation in the phenotype and homeostasis (27). An interesting proteomic analysis assay was performed using the mouse lung tumorigenic cell line E9-2, to understand how GJIC regulates tumorigenesis. Altered levels of protein disulfide isomerase, gelsolin-like protein, *α*-enolase, and aldolase A, were observed upon abrogation of mCx43 by transfection (28). In mouse models, the loss of one mCx43 allele alone resulted in a higher incidence of lung lesions (29). However, a study demonstrated that mCx43 loses its tumor-suppressing function in advanced carcinogenesis, thus, mCx43 is considered as a conditional tumor suppressor (29). Mice with one deleted allele of the mCx43 gene (mCx43^+/−^), mCx43 loss was associated with lung cancer aggression and a higher incidence of NSCLC-ad induced by the known carcinogen 7,12-dimethylbenzanthracene (24).

In the human NSCLC-ad A549 cell line, evidence supports that Cx is pro-tumorigenic particularly in cancer-associated fibroblasts, which accelerate the malignant progression of NSCLCs by forming Cx43-formed unidirectional GJIC from cancer-associated fibroblasts to A549 cells (30). The above-mentioned studies reveal a link between disruption of the steady state of Cx43 GJIC and lung tumorigenesis. This also indicates that maintaining the balance of the Cx43 steady state could be an important strategy for inhibition of lung tumorigenesis.

### Regulation of Cx43 During NSCLC Development

It is important to consider how Cx43 is regulated during lung cancer development and tumorigenicity advancement and metastasis. However, there are very limited published studies concerning the regulatory mechanism of Cx43 during lung cancer development. Hypoxia activates the P53/MDM2 axis and induces Cx43 internalization (31). Cx43 is moved from the membrane to cytoplasm, where it is degraded (31), and the lower Cx43 levels promote EMT, inducing proliferation and tumorigenicity in human NSCLC tissue and cells (31). In contrast, p38 MAPK activation and JNK inhibition increases Cx43-mediated cell-cell communication *via* in human lung neoplastic cells and this activation is induced by 4-phenyl-3-butenoic acid (32). Low Cx43 expression is also significantly associated with CpG island hypermethylation (CIH) in NSCLC. The level of CIH involved in poorly differentiated tumors and those caused by heavy smoking presented a weak Cx43 staining and ZO-1 or E-cadherin expression (33). Several studies indicated that Cx43 and E-cadherin are useful biomarkers in NSCLCs (22). Yeh and Hu proposed an explanation for conflicting reports on the functions of Cx43. They claimed that GJIC deficiency in the lung cancer A549 cells was mediated by oxidized beta-carotene, along with phosphorylation and abnormal positioning of Cx43 (34).

### Cx26, Cx31.1, Cx32, and Cx30.3 Functions in Lung Cancer Suppression

In addition to Cx43, other Cx subtypes are also involved in lung cancer cell proliferation, EMT, tumorgenicity, and metastasis. In hypoxia induced human pulmonary epithelial cells, low levels of Cx26 promoted EMT, inducing proliferation and tumorigenicity of cancer cells (31). In the A549 NSCLC-ad cell line expressing Cx26, the PI3K/AKT signaling pathway is involved in EMT (18). In human NSCLC cell lines, Cx31.1 expression was reduced and inversely correlated with lung cancer metastasis. Notably, Cx31.1 promoted the expression of cytokeratin, a marker of epithelial cell, and inhibited the expression of vimentin, a marker of mesenchymal cells (35). These data indicate that Cx31.1 induced limited shift from a mesenchymal phenotype towards an epithelial one, and that Cx31.1 in NSCLC can be anti-tumorigenic. The interesting link between Cx31.1 degradation by autophagy and tumor suppression in NSCLC H1299 cells (36) should be further investigated.

mCx32-knockout (KO) mice displayed increased tissue-specific sensitivity to radiation-induced tumorigenesis in the lung. mCx32 suppresses mouse lung tumorigenesis by altering the activation of the MAPK pathway in a p27 status-dependent manner (37).

In contrast to other Cxs, mCx30.3 promoted lung tumor growth and metastasis in a syngeneic mouse model, and its overexpression enhanced the sphere-forming ability and anchorage-independent growth of cancer cells (38). It is important to examine Cx30.3 in human tissue and cell lines to determine the utility of Cx30.3 as a diagnostic and prognostic biomarker for human lung cancer.

## Endothelial Cxs Expression and Implication in Lung Cancer Cell Extravasation

### Endothelial Cxs Promote Lung Cancer Cell Diapedesis

Cxs are expressed in endothelial cells. GJs regulate endothelial cell stiffness, a crucial physical characteristic related to several vascular pathologies. Tumor necrosis factor-α (TNFα) temporarily increases the stiffness of endothelial cells and is manipulated by the interaction of cells and rearrangement of the cytoskeleton (39). Additionally, an important Cx43 mediated signaling pathway (EGF-ERK1/2-FAK-RhoA-Rac1) was shown to determine the efficiency of A549 cell diapedesis. A549 cell-induced activation of human umbilical vein endothelial cells correlated with an increased abundance of Cx43 plaques on a co-culture of both cell types. Furthermore, loss of Cx43-GJIC in treatment using 18-*α*-glycyrrhetinic acid and siRNA caused a weakened activation of endothelial cells cell in the human umbilical vein (40). Pannexin1, which only forms hemichannels, is functionally notable; the permeability of vascular endothelial cells is regulated by Pannexin1 (41). Additionally, in retinal vascular endothelial cells, non-junctional Cx43 decreased the permeability of monolayer cells, and inhibited apoptosis mediated by high glucose (42). Cx-mediated endothelial cell dysfunction is essential for lung cancer metastasis, and endothelial cell dysfunction disrupts the cell integrity by causing inflammation. Consequently, integrity and permeability are to be protected by Cx in the endothelial cells. Aspirin alleviates endothelial cell dysfunction by inhibiting activation of the NLRP3 inflammasome in lipopolysaccharide-induced vascular injury (43).

Additionally, the human endothelial cell line, EAhy 926, treated with IL-1β/TNFα and high glucose, in Cx43 hemichannel inhibited and reduced ATP release (44). Further, homeostasis and intracellular communication were regulated by Cx37 and Cx40 by interaction with NOS (nitric oxide synthase) in the endothelial cells (45, 46). Importantly, compared to non-tumor lung samples, the higher NOS was found in the NSCLC-ad (47). However, effective strategies for identifying functional GJs between lung cancer cells and endothelial Cxs during the intravasation of lung cancer cells into blood vessels are limited.

### Cx43 in the Brain Endothelial Barrier Interacts With Lung Cancer Cells

Cx43 GJs are associated with hyperpermeability in the brain endothelial barrier. Cx43 is incorporated into the blood–brain barrier (BBB) junction complex, and the aberrantly increased Cx43 GJs regulate the permeability in a tight junction-dependent manner in the brain endothelial barrier (48). Additionally, Cx43 regulates the homeostasis of ions, pH, and permeability in the BBB. In a previous review, supporting evidence was described regarding the role of astroglial cells and Cxs in manipulating the permeability of BBB, initiated by infectious pathogenesis (49). HIV-infected astrocytes disrupt BBB integrity *via* a gap-junction-dependent mechanism due to endothelial apoptosis (50). cAMP activates cyclic nucleotide-gated channels, thereby inducing Ca^2+^ influx, leading to increased GJ coupling. Cyclic nucleotide-gated channels act as a physiological link that integrates GJ coupling into adenosine receptor-dependent signaling of BBB endothelial cells (51). Opening of the Cx43 hemichannel, polarized by acute ischemic stroke-mimicking conditions disrupted the transport function of BBB, and intracellular taurine and ATP were released in the BBB endothelial cells rat (TR-BBB13 cells) and human (hCMEC/D3 cells) origins (52). However, few studies have demonstrated GJ communication between lung cancer cells and endothelial Cxs during the extravasation of lung cancer cells and their crossing into the BBB.

**Figure 2:**
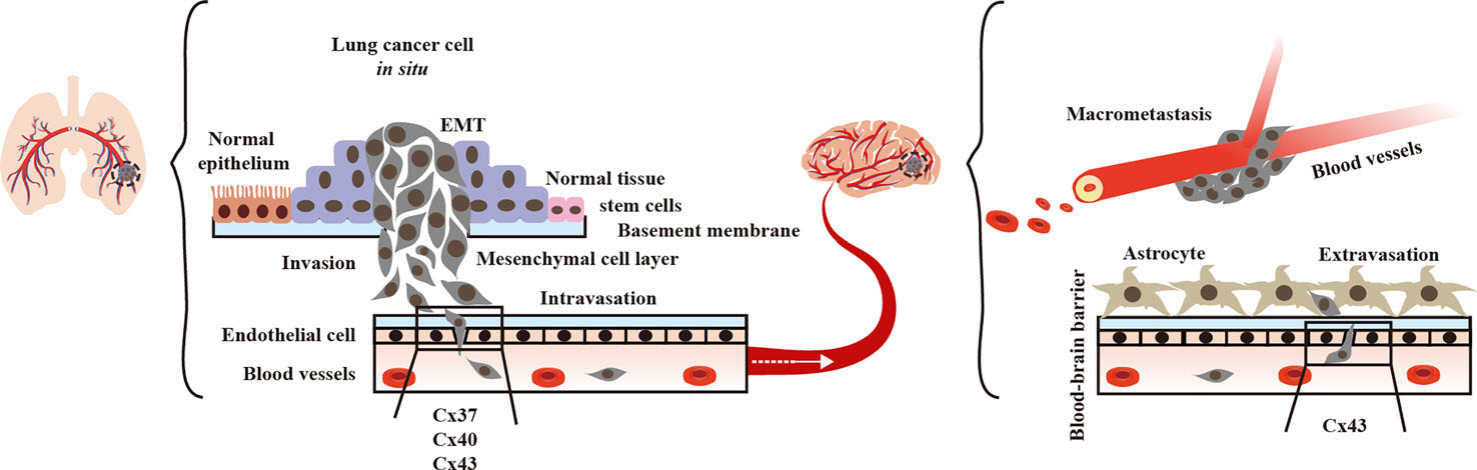
Endothelial Connexins (Cxs) allow lung cancer diapedesis. In blood vessels, endothelial Cx37, Cx40, and Cx43 contribute to the of lung tumor cells (40, 45, 46).

During extravasation, endothelial cell Cx37, Cx40, and Cx43 contribute to lung cancer cell diapedesis from vessels. Additionally, brain endothelial Cx43 helps lung tumor cells to traverse to the brain (**Figure 2**). Thus, we concentrated on the function of GJs interaction between lung tumor and the endothelium, as well as their effects on lung cancer brain metastasis.

## Astrocyte Cxs are Involved in Brain Metastasis of Lung Cancer

### Astrocyte Cxs Contribute to Lung Cancer Brain Metastasis

The interplay of the Cx43 junction channel with astrocytes and lung tumors promotes metastasis. Lung cancer cells require a growth-permissive microenvironment to survive, which is provided by the astrocytes (14). However, astrocytes can kill most infiltrated lung cancer cells (53) and thus, are emerging as essential regulators of brain metastasis (54). Brain metastatic cancer cells could form gap-junctional networks with the astrocytes and transfer cGMP to astrocytes, leading to the activation of the STING pathway, resulting in the production of interferon-α and TNF (55). Additionally, activating the MAPK/ERK signaling pathway promotes brain metastasis of lung cancer cells *via* a microRNA-330-3p-mediated mechanism (56). Subsequently, tumor cells and astrocytes are mutually stimulated in the microenvironment of brain metastasis by specific inflammatory cytokines, and this mutual relationship promotes lung cancer metastasis and its development in the brain (57). Brain-metastasized lung cancers show increased expression of nuclear beta-catenin, which increases Cx43 expression (58). Similarly, overexpression of Cx30.3 has been found to increase lung cancer metastasis (38). Furthermore, suppressors of GJs between lung A549 CSCs and astrocytes, such as AS602801, are an anti-CSC drug candidate to suppress brain metastasis (59).

### Astrocyte Cx43 Hemichannels Are Involved in Lung Cancer Brain Metastasis

Cx43 hemichannels, as well as GJIC in the astrocytes, may be involved in lung cancer brain metastasis. Astrocytes express high levels of Cx43, and the inhibition of cytokine-induced Cx43 hemichannels in astrocytes has a neuroprotective effect (60). Additionally, astrocyte Cx43 hemichannels increase the release of dickkopf-1 protein during HIV infection, thus contributing to brain pathogenesis observed in HIV-infected individuals (61). Notably, the activity of GJIC and hemichannel were differentially blocked by general anesthetics (propofol, ketamine, and dexmedetomidine), and had similar effects on neuronal hemichannels (62). In Parkinson’s disease, astrocytes Cx43 hemichannel activity regulated midbrain dopamine neuron degeneration in a glucocorticoid receptor-dependent manner. Increased Cx43 hemichannel activity was found *in vivo* in MPTP-intoxicated mice, and decreasing its activity by use of the hemichannel blocker TAT-Gap 19 peptide, increased dopamine neuron and microglial activation (63). Studies of osteocytes have shown that Cx43 hemichannels suppressed breast cancer growth and bone metastasis (12). Pro-inflammatory cytokines reduced Cx43 levels on the cell surface in activated microglia (64). Notably, NSCLC development was enhanced by circ_ZNF124, which was targeted by miR-337-3p directly to downregulate the JAK2/STAT3 signaling pathway (65), thereby providing indirect evidence of the role of the GJ–astroglial-STAT3 axis in lung cancer brain metastasis.

### Factors Affecting Astrocyte Cx43 Expression Are Associated With Lung Cancer Metastasis

Certain types of stress can affect Cx43 expression in astrocytes, and may be associated with lung cancer metastasis; however, direct evidence of this association is insufficient. Astrocytes Cx43 GJs ultrastructure changed in an oxygen–glucose deprivation mouse model. Oxygen–glucose deprivation-Cx43 metastasis-associated lung adenocarcinoma transcript I (MALAT1) may be related to lung cancer brain metastasis (66). Notably, MALAT1, the long noncoding RNA, protected human brain microvascular endothelial cells by inhibiting apoptosis induced by oxygen-glucose deprivation and reoxygenation (67). Norepinephrine is one of the most potent stimulators of tumor cell migration, and drives metastatic development of PC-3 human prostate cancer (68). It may be interesting to study the norepinephrine-Cx43-lung cancer brain metastasis axis. Notably, junctional channels between lung cancer cells and astrocytes induce resistance to chemotherapy (69). PC-14 NSCLC brain metastases were protected from chemotherapy by astrocytes *via* a mechanism of endothelin-dependent signaling (70).

**Figure 3:**
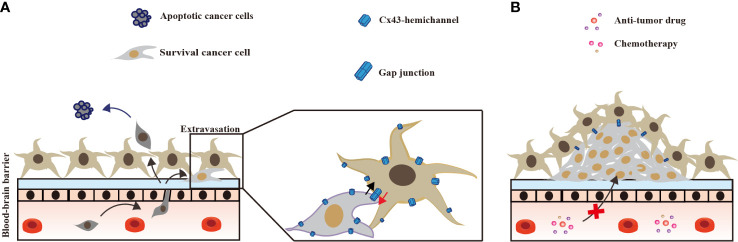
Astrocyte Cx43 interaction with brain metastases lung tumor. **(A)** At the occurrence of lung cancer brain metastasis, astrocytes kill most lung cancer cells that cross the blood–brain-barrier; few lung cancer cells survive form Cx43 gap junctions with astrocytes. **(B)** In the late stage of brain metastasis, Cx43 gap junctions between astrocytes and surviving lung cancer cells confer resistance against chemotherapy (53, 58).

In the brain, Cx43 in astrocytes inhibits invading lung cancer cells at an early stage, and forms GJs with surviving lung cancer cells. In the late stage, Cx43 GJ channels between lung cancer cells and astrocytes promote chemotherapy resistance (**Figure 3**).

## Concluding Remarks

A deeper understanding of the underlying mechanisms of Cx-mediated brain metastasis, and regrowth of lung tumors will help in improving strategies for prevention of lung cancer metastasis. Following primary tumor removal, the goal of systemic therapy should be to prevent relapse. However, adjuvant therapy agents target growing lung cancer cells rather than inhibiting metastasis. Cxs can function as both lung cancer suppressors and promoters depending on the isoforms, stages, and the type of lung cancer. Furthermore, endothelial Cxs contribute to lung cancer diapedesis and astrocyte Cxs contribute to the brain metastasis of lung cancer cells. Thus, using endothelial Cxs to build barriers against lung cancer diapedesis may be the best strategy for inhibiting the metastasis of lung cancer to the brain. There is a lack of research models and an appropriate lung cancer type to study all Cx isoforms, as well as tracking the entire process of lung cancer–brain metastasis. Furthermore, it is important to study lung cancer metastasis in the environmental context with potential lung carcinogens, such as coal combustion emissions. The findings of such investigations on the Cx isoforms and lung cancer types should be compared to global geographic variations, as reflected by the lung cancer rate in Xuanwei, China, which is the highest in the world (71, 72). Extrapolating knowledge from studies on Cxs from other cancer types, to lung cancer and brain metastasis may also foster the development of more effective therapeutic approaches.

There is a lack of therapeutics for lung cancers, but mimics peptides of Cxs currently under clinical trials for other diseases (73) and could be used to cancer therapeutics.

## Author Contributions

K-JL, Y-CH, and JJ designed and wrote the manuscript. C-XC constructed figures. J-PY collected references. EC proofread the manuscript. All authors contributed to the article and approved the submitted version.

## Funding

K-JL was supported by the Science and Technology Planning Project in Key Areas of Yunnan Province (202001BB050002) and the NSFC (31471823, 31772225, 31260448, 31060251) and has been awarded a State Scholarship Fund to pursue studies in the United States of America as a visiting scholar. Y-CH was supported by the NSFC (81960335), Key Project of International Cooperation of Science and Technology Innovation between Governments, and National Key Research and Development Plan of China (2016YEE0103400). JJ was supported by the Welch Foundation (AQ-1507) and NIH (CA196214).

## Conflict of Interest

The authors declare that the research was conducted in the absence of any commercial or financial relationships that could be construed as a potential conflict of interest.
